# Molecular Design and Mechanism Study of Non-Activated Collectors for Sphalerite (ZnS) Based on Coordination Chemistry Theory and Quantum Chemical Simulation

**DOI:** 10.3390/molecules29245882

**Published:** 2024-12-13

**Authors:** Xiaoqin Tang, Yilang Pan, Jianhua Chen, Ye Chen

**Affiliations:** 1 School of Chemistry and Chemical Engineering, School of Resources, Environment and Materials, Guangxi University, Nanning 530004, China; tangxiaoqin1850@163.com (X.T.); 2315302005@st.gxu.edu.cn (Y.P.); 2 State Key Laboratory of Featured Metal Materials and Life-Cycle Safety for Composite Structures, Guangxi University, Nanning 530004, China; 3 Guangxi Higher School Key Laboratory of Minerals Engineering, Guangxi University, Nanning 530004, China

**Keywords:** molecular design, interaction mechanism, sphalerite flotation, non-activated collector, coordination chemistry theory, quantum chemical simulation

## Abstract

Sphalerite flotation is generally achieved by copper activation followed by xanthate collection. This study aims to propose a design idea to find novel collectors from the perspective of molecular design and prove the theoretical feasibility that the collector can effectively recover sphalerite without copper activation. To address this, 30 compounds containing different structures of sulfur atoms and different neighboring atoms were designed based on coordination chemistry. Twelve potential collectors were screened, and their properties and interactions with a hydrated sphalerite (110) surface were evaluated. Compound **27 (C_2_H_4_S_2_^2−^)** showed the greatest reactivity, suggesting that the double-coordination structure of two sulfhydryl groups is an effective molecular structure for direct sphalerite flotation. The DFTB+ and MD results demonstrate that 1,2-butanedithiol (C_4_H_10_S_2_), having a similar coordination structure to compound **27**, has the potential to replace the traditional reagent scheme of sphalerite flotation. The strong reagent–surface interaction is attributed to the overlap of Zn 3d with S 3p orbitals, the most negative electrostatic potential, the relatively high E_HOMO_ and low average local ionization energy, and the eliminated steric hindrance effect. It is expected that this study can provide a design idea for the targeted design and development of novel reagents for complex sulfide ore flotation.

## 1. Introduction

Sphalerite (ZnS) is a typical sulfide ore and is one of the major sources of zinc metal, accounting for about 90% of zinc production [1]. Flotation is a mineral beneficiation method that utilizes the difference in hydrophilicity/hydrophobicity of different mineral surfaces to achieve the enrichment of target minerals [2,3,4]. The collector is one of the most important reagents in the mineral flotation process and can selectively interact on the mineral surface to make the mineral surface hydrophobic [5,6].

The relationship between the structure and performance of sulfide ore collectors has been fully explored by scholars. Among them, the “enantiomeric law” proposed by Wang [7] is widely recognized, i.e., when the polar group of the collector molecule and the target mineral have enantiomeric atoms (or groups of atoms), the collector is considered to be effective. Sulfur-bearing organic compounds occupy a very important position in sulfide ore flotation and can be broadly classified into three groups based on the different coordination structures of the sulfur atom and the different ligands of the sulfur atom, i.e., thiols (R–S–H), thioethers (R–S–R), and compounds containing the thiono group (–C(=S)–). According to the theory of coordination chemistry in mineral flotation [8,9], it is also known that neighboring atoms (e.g., N, O, and P atoms are the most common atoms in flotation reagents) have a non-negligible effect on their ligands, which, in turn, affects the nature of the collector and, hence, the interaction of the collector with the mineral surface. Therefore, the design of collector molecules needs to take into account not only the ligand but also the influence of neighboring atoms. Moreover, flotation reagents often consist of more than one functional group, so both monofunctional group coordination and multifunctional group coordination should be considered.

There have been many reports on the methods of molecular design of flotation reagents and the criteria for their reactivity [10]. For instance, Pradip and Rai (2003) [11] used molecular mechanics with the Universal Force Field (UFF) to model reagent molecules, mineral surfaces, and mineral–regent complexes, and the interaction energy (ΔE) was calculated to determine the most favorable mineral–regent interaction conformation. Wang et al. (2016) [12] used the CNDO/2 method to calculate the total energy changes (${\Delta E}_{T}^{RT}$) to reflect the adsorption and binding capacity of collectors with minerals. Apart from these, the criteria for designing collectors have also been built by adopting the conditional stability constant (CSC) [13,14], molecular mechanics (MM) [15,16], and the quantitative structure–activity relationship (QSAR) [17,18].

With the further development and refinement of quantum chemical theory in the last decade, especially the development of high-speed computer hardware, the first-principles approach based on density functional theory (DFT) has been widely applied to the calculation and simulation of solid surfaces [19] and further extended to the study of the interaction mechanism of surfactants at the solid–liquid interface, that is, the study of the interaction mechanism of the flotation reagent in the pulp [20]. Scholars have also proposed a series of collector design criteria using DFT, for instance, the Mulliken/Mulliken bonding population [21], density of states (DOS) [21], frontier molecular orbital energy [21], interaction distance [21], interaction energy [21], charge transfer [21], electrostatic potential [22], average local ionization energy (ALIE) [23], electronegativity [24], and atomic polarization rate [25]. A comprehensive evaluation of the above criteria can basically lead to a rational design of the molecular structure of the collectors and predict their flotation behavior and performance to some extent.

Sphalerite has poor natural floatability and is a sulfide mineral that is difficult to float. In industry, the flotation of sphalerite is generally achieved by copper activation followed by xanthate collection. The use of copper sulfate (as the activator) not only increases the operating cost but also brings difficulties to wastewater treatment. Moreover, copper sulfate is a very corrosive chemical that reduces equipment life. Collectors that float sphalerite without copper activation can significantly save costs, extend equipment life, and produce low-toxicity industrial wastewater, making it easier to meet environmental compliance.

The purpose of this study is to propose a design idea to discover a novel collector from the point of view of molecular design and prove its theoretical feasibility for the direct flotation of sphalerite in the absence of copper activation. To address it, 30 compounds containing three different structures of sulfur atoms (R–S–H, R–S–R, and –C(=S)–) and three different neighboring atoms (N, O, and P atoms) are designed based on the coordination chemistry theory of mineral flotation. Twelve sulfur-bearing collectors, containing similar coordination structures with the designed compounds, are screened, and their properties and interaction mechanisms with the fully hydrated sphalerite (110) surface are calculated by the density functional-based tight-binding (DFTB+) and molecular dynamics (MD) methods. It is expected that this study can uncover the molecular structure of efficient collectors for the copper-free activated flotation of sphalerite and provide a design idea for the targeted design and development of novel reagents for sphalerite flotation, and we expect that the design idea can be extended to the flotation of complex sulfide ores in the future.

## 2. Results and Discussion

### 2.1. Atomic Polarizability of Designed Collector Structures

In the soft and hard acids and bases (HSAB) theory proposed by Pearson [26], a hard base refers to molecules with a high charge density that are not easily polarized. Soft bases refer to molecules with a low charge density that are easily polarized and readily interact with the low, unoccupied orbitals of soft acids. The interaction between a hard base and a hard acid mainly forms electrovalence coordination, while the interaction between a soft base and a soft acid mainly forms covalent coordination. Metal ions in sulfide minerals are soft acids, and commonly used sulfur-bearing collectors are soft bases; hence, the interaction between sulfide minerals and sulfur-bearing collectors belongs to the interaction between soft acids and soft bases, mainly covalent coordination. Therefore, by discussing the covalency of sulfur-bearing collectors, effective collector structures for sphalerite flotation can be screened out.

Atomic polarizability can be used to measure the electron deformability of bonded atoms [27]. It is generally believed that the greater the polarizability, the greater the deformation ability of the electron cloud, resulting in an increase in covalent coordination ability.

The atomic polarizabilities of the S atoms in the 30 designed compounds are listed in Table 1. As shown, the atomic polarizabilities of the two single-bonded S atoms in compound **27** are the largest (32.285 a.u.), demonstrating the greatest covalency among these 30 designed compounds. In addition, compounds **4**, **5**, **7**, **9**, **22**, and **23** have relatively large atomic polarizabilities and can also be considered potentially effective collector structures for copper-free activated sphalerite flotation. From the perspective of molecular structure, these compounds all have similar coordination structures of sulfur atoms and neighboring atoms to traditional sulfur-bearing collectors (such as xanthate, diethyldithiocarbamate, thiourea, dithiophosphate, O-isopropyl ethylthiocarbamate, and thiol), which also confirms their collection ability for sulfide minerals from the side.

Based on the atomic polarizability analysis of the above 30 designed compounds, 11 traditional sulfur-bearing collectors containing the effective coordination structure in compounds **4**, **5**, **7**, **9**, **22**, and **23** were selected, and a new collector containing a double-coordination structure of two sulfhydryl groups in compound **27** was designed. Subsequently, DFTB+ and MD were used to investigate the interaction mechanisms of these 12 collectors on the fully hydrated sphalerite (110) surface.

### 2.2. Adsorption of Screened Collectors on Fully Hydrated Sphalerite Surface

Flotation occurs in an aqueous environment. Our previous studies have shown that the presence of water molecules affects not only the interaction between the mineral surface and water molecules but also the interaction of the mineral surface with the reagent [28]. However, in the existing DFT studies involving the adsorption of reagents on the sphalerite surface, it either did not adsorb water molecules or adsorbed only a small number of water molecules, which is insufficient to reflect the true aqueous environment of flotation.

**Figure 1:**
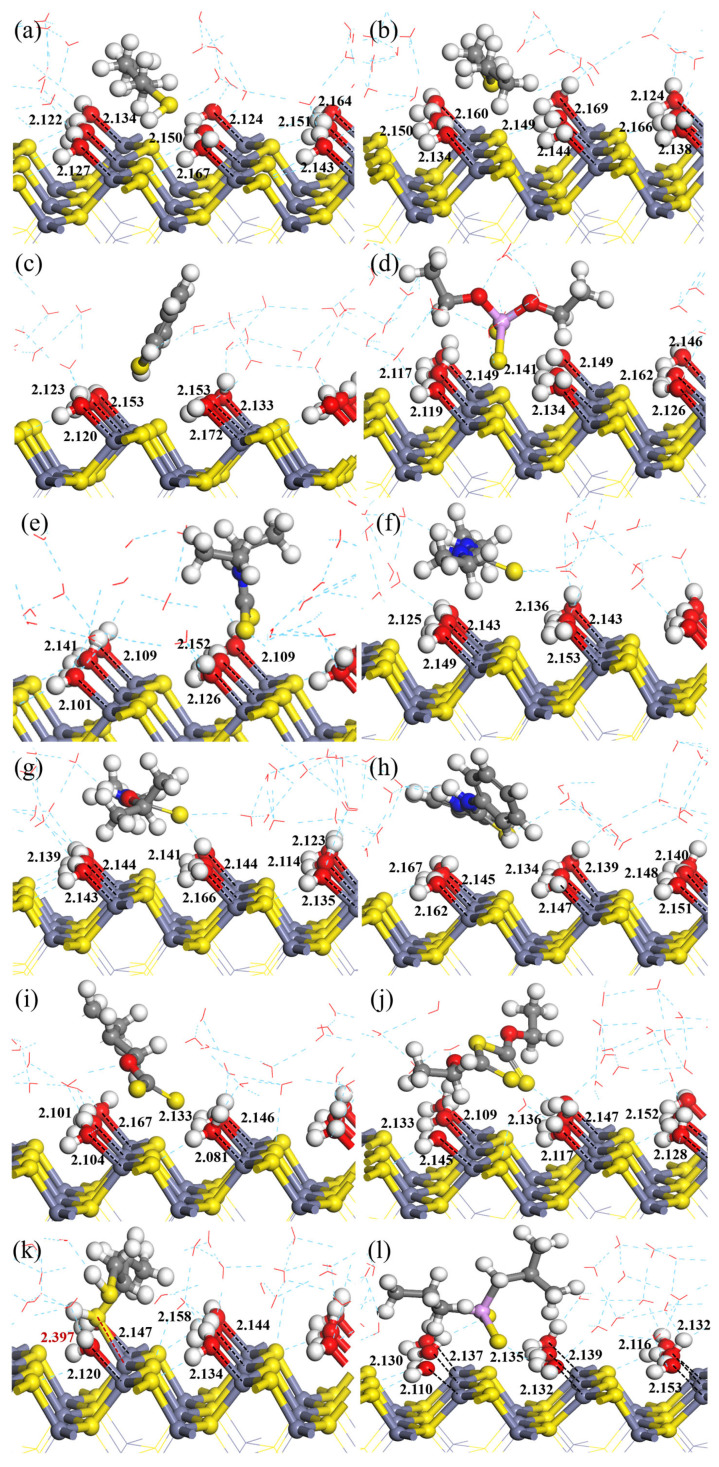
Optimized geometry configurations of 12 collectors adsorbed on the sphalerite (110) surface in the presence of 3 layers of water molecules (unit: Å): (**a**) 1-BT, (**b**) DS, (**c**) TP, (**d**) DTP, (**e**) DDTC, (**f**) DET, (**g**) ETC, (**h**) DPT, (**i**) BX, (**j**) EDX, (**k**) 1,2-BDT and (**l**) DTPP.

The sphalerite surface is readily hydrated; therefore, in order to better reflect the real flotation aqueous environment, the adsorptions of 12 screened collectors on the sphalerite (110) surface in the presence of three layers of water molecules (viz., 27 water molecules) were simulated to investigate the influence of surface hydration on the adsorption behavior of collectors, and the optimized geometry configurations are shown in Figure 1. Table 2 lists the corresponding Zn–S and Zn–O interaction distances and adsorption energies.

As shown in Figure 1a–j,l, the O atoms in the first layer of water molecules are bonded with the surface Zn atoms, with collector molecules being distributed away from the surface. Their average Zn–O interaction distances and adsorption energies are within the range from 2.122 Å to 2.148 Å and −21.41 kJ/mol to −29.52 kJ/mol, respectively, demonstrating that on the hydrated co-adsorption surface, water adsorption is more favorable than collector adsorption, and water molecules are weakly adsorbed on the hydrated surface in the presence of collector 1-BT, DS, TP, DTP, DDTC, DET, ETC, DPT, BX, EDX, or DTPP. Among these 11 collectors, the adsorption energy of BX on the collector and water co-adsorbed sphalerite surface is the most negative (−29.52 kJ/mol). This may explain why most sphalerite flotation practices adopt xanthates as collectors rather than other conventional sulfur-bearing collectors. Although BX is the relatively most effective sphalerite collector among the 11 collectors, their adsorption energies are all within the range of physisorption, and the use of BX as a sphalerite collector always requires the activation of copper ions, which suggests that none of the 11 collectors is sufficiently effective to recover sphalerite without activation by copper ions.

However, as shown in Figure 1k, one S atom in 1,2-BDT interacts with one surface Zn atom with an interaction distance of 2.397 Å, and the remaining surface Zn atoms interact with the O atoms in eight water molecules with an average interaction distance of 2.141 Å, and the corresponding adsorption energy is −99.20 kJ/mol, indicating that on the hydrated co-adsorption surface, 1,2-BDT adsorption is more favorable than water adsorption, and 1,2-BDT is more strongly adsorbed on the hydrated sphalerite surface. The adsorption energy is much more negative than that of the remaining 11 collectors and within the range of chemisorption. Furthermore, the interaction distance and adsorption energy are comparable to the copper-activated model using BX as the collector reported in the literature [29], demonstrating that 1,2-BDT is sufficiently effective in recovering sphalerite without copper activation.

From the adsorption configuration and adsorption energy perspective, it can be concluded that among these 12 collectors, 1,2-BDT is the most effective collector for sphalerite flotation, and it seems to own the potential to recover sphalerite in the absence of copper activation.

**Figure 2:**
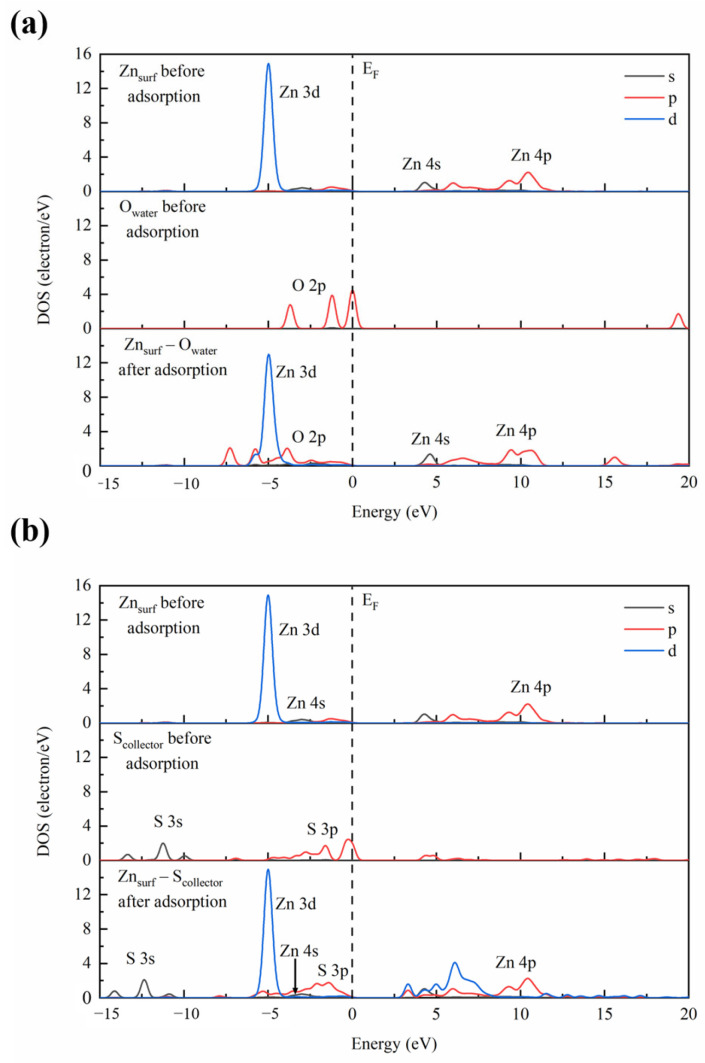
DOSs of the surface Zn atoms and (**a**) water O (**b**) collector S atoms and their interactions on the sphalerite (110) surface.

The densities of states (DOSs) of the collector S atoms (denoted as S_collector_), water O atoms (denoted as O_water_), and surface Zn atoms (denoted as Zn_surf_) before adsorption and the Zn–O/Zn–S interactions after adsorption are illustrated in Figure 2. As shown in Figure 2, the Zn 3d orbitals show greater DOSs than the Zn 4s orbitals near the Fermi level (E_F_), indicating Zn 3d orbitals make a greater contribution to the reactivity of atoms on the sphalerite surface. The DOSs of the Zn 3d orbitals are distributed in the deep energy level from −5.89 eV to −4.10 eV, demonstrating that the Zn 3d state is relatively inactive. As illustrated in Figure 2a, after co-adsorption, the DOSs of the O 2p orbitals shift toward the deeper energy level and overlap with the Zn 3d orbitals within a narrow energy level from −6.07 eV to −4.27 eV, with the localizability of the Zn 3d orbitals’ DOSs remaining almost the same. Similarly, as shown in Figure 2b, the collector–mineral interaction shifts the DOSs of the S 3p orbitals slightly toward the deeper energy level and allows them to overlap with the Zn 3d orbitals within a relatively narrower energy level from −5.70 eV to −4.28 eV, with the localizability of the Zn 3d orbitals’ DOSs remaining unchanged. It is worth noting that the overlap of the Zn 3d orbitals with the S 3p orbitals is also observed within a relatively broader energy level from 2.88 eV to 8.35 eV, with their DOSs becoming more delocalized, which suggests that the interaction between S_collector_ and Zn_surf_ atoms is greater than that between O_water_ and Zn_surf_ atoms.

Hence, it can be concluded that on the 1,2-BDT and water co-adsorbed sphalerite surface, the adsorption of collector 1,2-BDT is more favorable. The adsorption of 1,2-BDT repels one water molecule that is supposed to be adsorbed onto the surface away from the surface, hence improving the hydrophobicity of the surface. Consequently, the surface becomes more favorable for collector adsorption.

### 2.3. Radial Distribution Function (RDF) Analysis Based on MD Simulations

In the existing DFT studies involving the adsorption of reagents on the sphalerite surface, the temperature is generally set to absolute zero by default, which fails to simulate the actual flotation temperature. Hence, it is of great significance to construct a model that can reflect the real flotation temperature, as well as the aqueous environment and the interactions between water, reagents, and minerals at the solid–liquid interface during sphalerite flotation.

**Figure 3:**
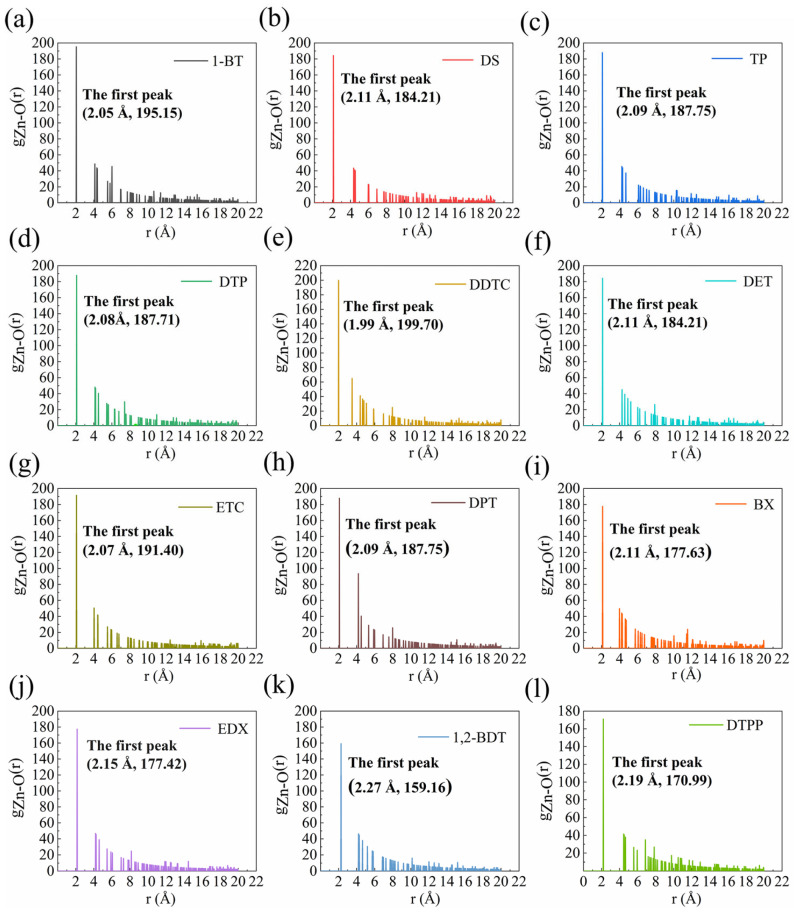
RDFs of Zn atoms on the sphalerite (110) surface with respect to O atoms in water molecules in the presence of 12 collectors: RDFs of (**a**) 1-BT, (**b**) DS, (**c**) TP, (**d**) DTP, (**e**) DDTC, (**f**) DET, (**g**) ETC, (**h**) DPT, (**i**) BX, (**j**) EDX, (**k**) 1,2-BDT and (**l**) DTPP.

The radial distribution function (RDF) for the water molecules on the sphalerite (110) surface was derived from MD simulations in which 12 collector molecules and three layers of water molecules were placed on the mineral surface. The distribution probability of a β-particle with respect to a given α-particle when the distance is *r* is defined as the RDF, *g*(*r*). The system temperature was set to 298.15 K to simulate the actual flotation temperature. Figure 3 illustrates the RDFs of O_water_ atoms relative to Zn_surf_ atoms (viz., the distances of the water molecules from the mineral surface) to characterize the adsorption characteristics of the water molecules on the surface as well the hydrophobicity of the surface, and it presents the locations of the first RDF peaks and their first peak intensities when the 12 collectors and water are co-adsorbed.

As shown in Figure 3, the water molecules are distributed at >1.99 Å above the collector and water co-adsorbed surfaces. More specifically, the first peak of the 1,2-BDT and water co-adsorbed configuration (as shown in Figure 3k) occurs at 2.27 Å above the surface, which is greater than that of the remaining 11 collectors and water co-adsorbed configurations, demonstrating that the 1,2-BDT and water co-adsorbed surface is the most hydrophobic. The first peak intensity of the 1,2-BDT and water co-adsorbed configuration is 159.16, which is significantly smaller than that of the remaining 11 configurations, indicating that the distribution of the first layer of water molecules on the 1,2-BDT and water co-adsorbed surface is less ordered. As the distance *r* increases, the decay of the peaks flattens out, and it becomes increasingly difficult to observe distinct peaks, suggesting that the distribution of water molecules away from these surfaces is becoming increasingly disordered.

In conclusion, the above results demonstrate that although the distribution of water molecules on the 1,2-BDT and water co-adsorbed sphalerite surface is less ordered than that of the remaining 11 sulfur-bearing collectors, the surface is the most hydrophobic. Therefore, it is reasonable to assume that 1,2-BDT is the most effective sphalerite collector among the 12 collectors.

The above calculation results confirm that 1,2-BDT is the most effective among the 12 collectors screened, and its potential for copper-free activated flotation of sphalerite has been discovered. Herein, the interaction mechanisms of the sphalerite surface with 1,2-BDT and the remaining 11 collectors are further interpreted from a more theoretical perspective.

As mentioned in Section 2.1, the interaction between sphalerite and a sulfur-bearing collector belongs to the interaction between a soft acid and a soft base and is dominated by covalent coordination. Therefore, the HOMO orbital energy (E_HOMO_), electrostatic potential, and ALIE are further used to discuss the covalent coordination ability between the 12 collectors and the sphalerite surface.

Frontier molecular orbitals were proposed by Fukui [30] in 1952. In the soft acid–soft base reaction, the higher the HOMO orbital energy of the base (viz., the sulfur-bearing collector), the smaller the energy difference between the LUMO orbital energy of the acid (viz., the sphalerite surface) and the HOMO orbital energy of the base, and the easier the electrons can be transferred, and hence, the easier it is to form covalent bonding, that is, the stronger the covalent coordination ability of the base.

The negative electrostatic potential indicates that the electrostatic potential here is dominated by the charge of the electron [31]. It can be considered that the more negative the electrostatic potential, the easier it is to attract the attack of the electrophilic reagent and the stronger the covalent coordination.

ALIE represents the ionization energy of an electron at a local location and has a wide range of applications [32], and its most important application is the prediction of reaction sites. It is generally believed that the smaller the ALIE value, the weaker the electron-binding ability, and the more likely covalent coordination is to occur.

The E_HOMO_, electrostatic potential, and ALIE values of 12 screened collectors are presented in Table 3. As shown, the electrostatic potential of 1,2-BDT is the most negative, with the E_HOMO_ and ALIE being the third after DET and 1-BT. The E_HOMO_ of DET (−2.667 eV) and 1-BT (−4.517 eV) is higher than that of 1,2-BDT (−4.544 eV), and the ALIE of DET (4.383 eV) and 1-BT (4.934 eV) is slightly smaller than that of 1,2-BDT (5.165 eV). However, the electrostatic potential of 1,2-BDT (−1034.500 kJ/mol) is significantly more negative than that of DET (−637.591 kJ/mol) and 1-BT (−584.126 kJ/mol), indicating the strongest covalent coordination ability of 1,2-BDT.

Moreover, on the clean sphalerite (110) surface, after surface relaxation, one Zn atom coordinates with three S atoms to generate a planar triangular structure. Due to the large atomic radius of the S atom, the S atom on the sphalerite surface spatially hinders the Zn atom, resulting in the steric hindrance effect [20]. To overcome this issue, the surface Zn atoms have to move upward by at least 0.2 Å [20]. Copper activation is achieved by substituting surface Zn atoms with Cu atoms, which shifts the surface Cu atoms upward by 0.4 Å [20] and, consequently, eliminates the steric hindrance effect. However, when using 1,2-BDT as the collector, the Zn–S bonding effect shifts one surface Zn atom upward by 0.3 Å, which is also greater than the minimum displacement required to overcome the steric hindrance effect on the clean sphalerite (110) surface. Therefore, it is reasonable to conclude that among the 12 collectors screened, 1,2-BDT has the greatest potential to achieve the direct flotation of sphalerite in the absence of copper activation, and the double-coordination structure of two sulfhydryl groups in its molecular structure is the most effective.

**Figure 4:**
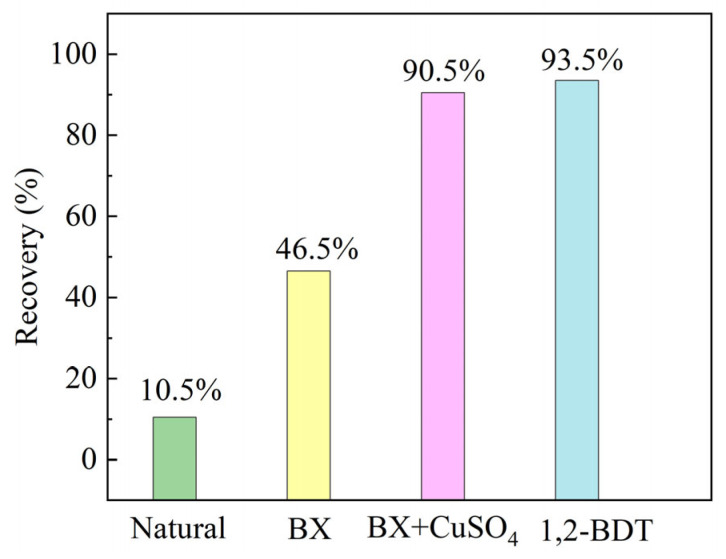
Flotation recoveries of natural sphalerite (pH = 7) and sphalerite collected by BX (pH = 7; [BX] = 1 × 10^−4^ mol/L), BX + CuSO_4_ (pH = 7; [BX] = 1 × 10^−4^ mol/L and [CuSO_4_] = 1 × 10^−4^ mol/L), and 1,2-BDT (pH = 7; [1,2-BDT] = 1 × 10^−4^ mol/L).

Furthermore, a set of single mineral flotation tests were conducted to verify the practical feasibility of the selected collector, 1,2-BDT, to recover sphalerite in the absence of copper sulfate activation. The details of the sphalerite sample and the flotation process can be found in the Supplementary Material. Figure 4 demonstrates the flotation recoveries of natural sphalerite and sphalerite collected by BX, BX + CuSO_4_, and 1,2-BDT. As shown in the figure, the natural floatability of the sphalerite sample used is 10.5%, suggesting the inferior natural floatability of this sphalerite sample. At pH = 7, using 1 × 10^−4^ mol/L BX as the collector, the sphalerite recovery increases to 46.5%, and with the addition of 1 × 10^−4^ mol/L copper sulfate and 1 × 10^−4^ mol/L BX, the sphalerite recovery further increases to 90.5%. However, in the absence of copper sulfate, the optimal recovery of sphalerite recovered by 1 × 10^−4^ mol/L 1,2-BDT reaches 94.5%, demonstrating that 1,2-BDT is effective in the direct recovery of sphalerite, and the results using 1,2-BDT as the collector in the absence of copper sulfate activation are comparable to the results using BX as the collector and copper sulfate as the activator. Therefore, the practical value of this research can be verified.

## 3. Materials and Methods

### 3.1. Computational Methods and Models

#### 3.1.1. Molecular Design and Screening of Potential Collector Structures

For the optimization of the molecular structure of potential collectors and the calculation of their properties, the Gaussian 16 (c.01 version) [33] and Multiwfn (3.8 version) [34] software were used in this study. For the geometrical structure optimization of a single-molecule structure, the B3LYP general function has good performance in terms of the computational cost, applicability, and computational time [35], and thus, the computational level of B3LYP/6-311+G** was selected. Considering that mineral flotation is usually carried out in an aqueous solution, the SMD solvation model was adopted, and water was selected as the solvent.

Table 4 presents the molecular and steric configurations of 30 designed compounds for the copper-free activated flotation of sphalerite, taking into account the effect of three coordination structures of the sulfur atom, three different neighboring atoms, and multifunctional group coordination.

#### 3.1.2. Properties and Mechanism Study of Screened Collector Structures

The DFTB+ module in the Materials Studio (MS) software (2019 version) was used to calculate the interaction between the sphalerite surface and the screened copper-free activated sphalerite collector containing the effective molecular structures. This module is a quantum chemical calculation method based on the second-order expansion of the Kohn–Sham total energy in DFT via charge density fluctuations [36]. It not only retains the accuracy of DFT but also has the efficiency of tight-binding methods, with the main advantage of being able to achieve calculations for large systems containing hundreds of atoms at a greatly reduced computational cost while maintaining accuracy [37].

The molecular formula and steric configurations of the 12 collectors containing the screened coordination structures are presented in Table 5. In the aqueous environment, both hydrogen bonding and water–surface interactions are involved in the adsorbed water molecules. However, based on our past studies [1], neither the isolated water molecule model nor the competitive adsorption model involving multiple water molecules can accurately reflect the hydration of the mineral surface. Therefore, the model involving excess water molecules, i.e., three layers of water molecules, at the surface was established to understand the competition between the hydrogen bonding and water–surface interactions. The models for the geometry optimization of the mineral surface and the adsorption of 12 different collectors and 3 layers of water molecules involve a relatively large system, owning at most 218 atoms (viz., diisobutyl dithiophosphinate and 27 water molecules co-adsorbed on the sphalerite surfaces, Zn_54_C_8_H_72_O_27_S_56_P), and thereby, these computations were carried out via the DFTB+ module in the MS software. Before adsorption, the surface model, the water molecule, and the collector molecules were geometrically optimized. Then, a layer of water molecules and a collector molecule were placed on the sphalerite surface to perform geometrical optimization again. To ensure the accuracy of the absorbing energy, the water molecules were placed layer by layer, and the geometrical optimization was performed after every placement of water.

The Monkhorst–Pack scheme was adopted for the Brillouin zone sampling, with the k-point gridding being set as 1 × 2 × 1 [38]. During the geometry optimization and energy calculation, the energy tolerance, the maximum force tolerance, and the maximum displacement tolerance were set as 0.05 kcal/mol, 0.5 kcal/mol/Å, and 0.01 Å, respectively. The self-consistent charges (SCCs) were adopted in all calculations, with the SCC tolerance and eigensolver being set as medium and divide and conquer, respectively. The smearing was 0.0025 Ha. Due to the presence of hydrogen bonds and other weaker intermolecular forces between collectors and water molecules during adsorption, dispersion correction (DFT-D3) was applied to all calculations.

The space group of sphalerite is *F*$\bar{4}$*3m* with an isometric structure [39]. After the geometry optimization of the bulk structure, the calculated lattice parameters were *a* = *b* = *c* = 5.435 Å, which were close to the experimental value of *a* = *b* = *c* = 5.414 Å [40]. In the optimized bulk structure of sphalerite, each S (Zn) atom coordinated with four Zn (S) atoms, forming a tetrahedral structure. Then, the cleavage surface was constructed based on the optimized bulk structure. The surface energy of the sphalerite (110) surface was tested to be the lowest, suggesting a relatively stable structure, and thus, this surface was selected in this study. After testing the slab thickness, a (3 × 3 × 1) supercell with six atomic layers (cell formula is Zn_54_S_54_) was obtained, with the top two atomic layers being allowed to relax and the bottom four atomic layers being fixed. This supercell was separated by a 20 Å vacuum layer to avoid mirror effects. The dimensions of the supercell were 16.304 × 11.529 × 29.607 Å^3^, which was sufficient for the subsequent adsorption of water molecules and collectors. The valence electron configurations mentioned in this study were O 2s^2^2p^4^, S 3s^2^3p^4^, and Zn 3d^10^4s^2^.

Before adsorption, the geometry of the water molecule and 12 collector molecules was optimized in a 15 × 15 × 15 Å^3^ cubic cell, with the parameter setting being the same as when geometrically optimizing the sphalerite unit cell. The following Equation (1) can be used to calculate the adsorption energies of the collector/water molecules on the sphalerite (110) surface:

(1) $$\Delta E_{ads}=E_{surf+adsorbate}-E_{surf}-E_{adsorbate}$$

where Δ*E_ads_* denotes the adsorption energy (kJ/mol) of the adsorbates adsorbed on the sphalerite (110) surface; *E_surf+adsorbate_* represents the total energy of the sphalerite (110) surface adsorbing the screened collectors and water molecules after optimization (kJ/mol); and *E_surf_* and *E_adsorbate_* are the energies of the sphalerite (110) surface and the screened collectors/water molecules after optimization (kJ/mol), respectively.

In order to better discuss the collector–mineral interaction mechanism from a theoretical perspective, the energy of the highest occupied molecular orbital (HOMO), electrostatic potential, and ALIE were also calculated at the computational level of B3LYP/6-311+G** using the Gaussian 16 and Multiwfn software. The calculation parameters remained the same.

### 3.2. Molecular Dynamics (MD) Simulations

MD simulations were performed to investigate the influence of temperature on the collector adsorption on the hydrated sphalerite (110) surface. The DFTB+ module in the MS program was also adopted to carry out the simulation calculations. The system temperature was controlled by a Noséthermostat, with the temperature being set as 25 °C (viz., 298.15 K), in order to simulate the real flotation temperature. The time step was 1.0 fs, and the total simulation time was 10.0 ps. The canonical (NVT) ensemble was used all the time. The remaining parameters were set to the default values. Although the conformational search of the collector molecular structure was not performed before the MD calculations, the geometrically optimized collector molecules are considered structurally stable, and their energies are supposed to be the lowest.

## 4. Conclusions

In this study, 30 S-bearing compounds were designed based on the coordination chemistry theory of mineral flotation. Twelve potential collectors were screened, and their interactions with the sphalerite (110) surface were calculated by the DFTB+ and MD methods in order to reveal the theoretical feasibility of using the designed novel collector to replace the traditional reagent scheme of sphalerite flotation. The main conclusions are as follows:

I. Compound **27** shows the largest atomic polarizability, demonstrating the greatest covalency among the 30 compounds designed, which can indicate that the double-coordination structure of two sulfhydryl groups might be an effective molecular structure for sphalerite flotation.
II. Both the DFTB+ and MD calculation results confirm that the adsorption configurations and interaction energy of 1,2-BDT, having a similar coordination structure to compound **27**, on the fully hydrated sphalerite surface are comparable to the copper-activated model using BX as the collector, suggesting that 1,2-BDT owns the potential to directly collect sphalerite in the absence of copper activation.
III. The strong 1,2-BDT–sphalerite interaction can be attributed to the overlap of Zn 3d orbitals with S 3p orbitals, the most negative electrostatic potential, the relatively high E_HOMO_ and low ALIE, and the eliminated steric hindrance effect.

In the follow-up study, the flotation behavior and interaction mechanism of a series of dithiol collectors on the copper-free activated flotation of sphalerite will be further studied from both experimental and theoretical perspectives so as to realize the efficient flotation of sphalerite. It is expected that this study can provide a design idea for the targeted design and development of new reagents for sphalerite flotation and extend its application for the flotation of complex sulfide ores in the future.

## Figures and Tables

**Table 1:** Atomic polarizability of the S atoms of 30 designed compounds.

Compound	Atomic Polarizability of −S (a.u.)	Atomic Polarizability of =S (a.u.)	Compound	Atomic Polarizability of −S (a.u.)	Atomic Polarizability of =S (a.u.)
**1**	20.605	/	**16**	20.490	/
**2**	23.688	23.688	**17**	20.206	/
**3**	17.804	20.031	**18**	17.755	19.981
**4**	24.422	22.506	**19**	17.787	20.334
**5**	23.977	23.973	**20**	18.288	/
**6**	23.666	23.667	**21**	18.210	/
**7**	/	21.611	**22**	22.842	22.842
**8**	/	19.721	**23**	/	20.702
**9**	24.155	24.155	**24**	18.829	/
**10**	28.336	/	**25**	21.562	/
**11**	28.172	/	**26**	21.397	/
**12**	25.538	/	**27**	32.285	/
**13**	18.643	/	**28**	19.824	/
**14**	18.679	/	**29**	/	19.598
**15**	20.697	/	**30**	17.912	20.696

**Table 2:** Zn–S and Zn–O interaction distances and adsorption energies of 12 collectors adsorbed on the sphalerite (110) surface in the presence of 3 layers of water molecules.

Collector	Zn–S Interaction Distance (Å)	Zn–O Interaction Distance (Å)	Adsorption Energy (kJ/mol)
1-BT	/	2.122–2.167 (Average: 2.142)	−28.01
DS	/	2.124–2.169 (Average: 2.148)	−27.22
TP	/	2.120–2.172 (Average: 2.142)	−27.04
DTP	/	2.117–2.162 (Average: 2.138)	−27.08
DDTC	/	2.101–2.152 (Average: 2.123)	−25.13
DET	/	2.125–2.153 (Average: 2.142)	−26.26
ETC	/	2.114–2.166 (Average: 2.139)	−26.44
DPT	/	2.134–2.167 (Average: 2.148)	−21.41
BX	/	2.081–2.167 (Average: 2.122)	−29.52
EDX	/	2.109–2.152 (Average: 2.133)	−25.59
1,2-BDT	2.397	2.120–2.158 (Average: 2.141)	−99.20
DTPP	/	2.110–2.153 (Average: 2.132)	−24.13

**Table 3:** E_HOMO_, electrostatic potential, and ALIE values of 12 screened collectors.

Reagent	E_HOMO_ (eV)	Electrostatic Potential (kJ/mol)	ALIE (eV)
1-BT	−4.517	−584.126	4.934
DS	−5.986	−122.322	6.729
TP	−4.680	−547.349	5.562
DTP	−5.524	−552.842	6.594
DDTC	−4.599	−635.883	5.975
DET	−2.667	−637.591	4.383
ETC	−6.041	−238.141	6.721
DPT	−5.878	−201.594	6.908
BX	−5.061	−587.701	6.058
EDX	−6.503	−138.018	7.875
1,2-BDT	−4.544	−1034.500	5.165
DTPP	−5.197	−602.037	6.127

**Table 4:** Molecular formulas and steric configurations of 30 designed compounds.

Compound	Molecular Formula	Steric Configuration	Compound	Molecular Formula	Steric Configuration
**1**	C_2_H_5_S_2_^−^	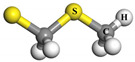	**2**	C_2_H_3_S_2_^−^	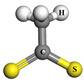
**3**	C_3_H_6_S_2_	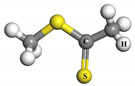	**4**	C_2_H_3_OS_2_^−^	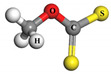
**5**	C_3_H_6_NS_2_^−^	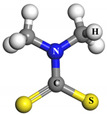	**6**	C_5_H_12_PS_2_^−^	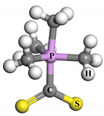
**7**	C_3_H_8_N_2_S	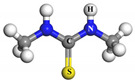	**8**	C_3_H_6_O_2_S	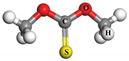
**9**	C_2_H_6_PS_2_^−^	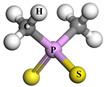	**10**	CH_3_OS^−^	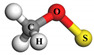
**11**	C_2_H_6_NS^−^	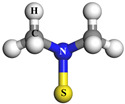	**12**	C_4_H_12_PS^−^	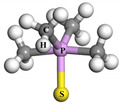
**13**	C_2_H_6_OS	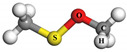	**14**	C_3_H_9_NS	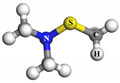
**15**	C_5_H_14_PS_2_^−^	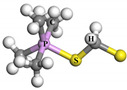	**16**	CH_3_OS_2_^−^	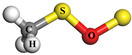
**17**	CH_4_NS_2_^−^	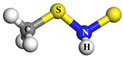	**18**	C_3_H_6_OS_2_	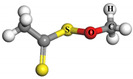
**19**	C_4_H_9_NS_2_	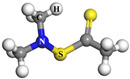	**20**	C_3_H_6_O_2_S	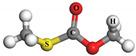
**21**	C_4_H_9_NOS	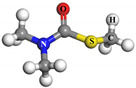	**22**	C_2_H_6_PO_2_S_2_^−^	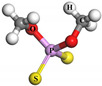
**23**	C_3_H_7_NOS	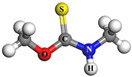	**24**	C_2_H_7_NS	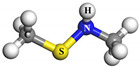
**25**	C_2_H_6_NS_2_^−^	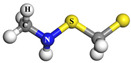	**26**	C_2_H_6_NS_2_^−^	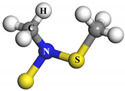
**27**	C_2_H_4_S_2_^2−^	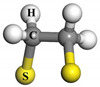	**28**	C_3_H_8_S_2_	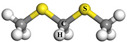
**29**	C_4_H_6_S_2_	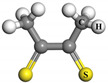	**30**	C_3_H_7_NS_2_	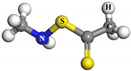

**Table 5:** Molecular formulas and steric configurations of 12 collectors containing the screened coordination structures.

Reagent	Molecular Formula	Description	Steric Configuration
1-Butanethiol (1-BT)	C_4_H_10_S	Single coordination structure of –SH	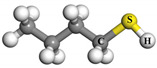
Diethyl sulfide (DS)	C_4_H_10_S	Single coordination structure of –S–	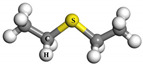
Thiophenol (TP)	C_6_H_6_S	Single coordination structure of –SH	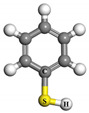
O,O-diethyl dithiophosphate (DTP)	C_4_H_10_O_2_PS_2_^−^	Similar coordination structure with compound **22**	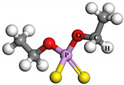
Diethyldithiocarbamate (DDTC)	C_5_H_10_NS_2_^−^	Similar coordination structure with compound **5**	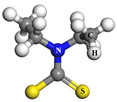
N,N’-diethylthiourea (DET)	C_5_H_12_N_2_S	Similar coordination structure with compound **7**	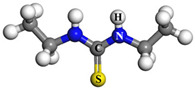
O-isopropyl ethylthiocarbamate (ETC)	C_6_H_13_NOS	Similar coordination structure with compound **23**	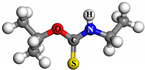
N,N′-diphenylthiourea (DPT)	C_13_H_12_N_2_S	Similar coordination structure with compound **7**	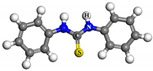
Butyl xanthate (BX)	C_4_H_9_OCSS^−^	Similar coordination structure with compound **4**	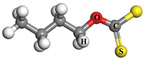
Ethyl dixanthate (EDX)	C_4_H_10_O_2_C_2_S_4_	Similar coordination structure with compound **4**	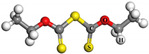
1,2-butanedithiol (1,2-BDT)	C_4_H_10_S_2_	Similar coordination structure with compound **27**	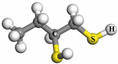
Diisobutyl dithiophosphinate (DTPP)	C_8_H_18_PS_2_^−^	Similar coordination structure with compound **9**	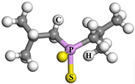

## Data Availability

Data is contained within the article or Supplementary Materials.

## Supplementary Materials

The following supporting information can be downloaded at: https://www.mdpi.com/article/10.3390/molecules29245882/s1, Figure S1: XRD analysis results of sphalerite.
