# Supplementary material for: Molecular Design and Mechanism Study of Non-Activated Collectors for Sphalerite (ZnS) Based on Coordination Chemistry Theory and Quantum Chemical Simulation
Source: Molecules. 2024 Dec 13;29(24):5882. doi: 10.3390/molecules29245882 (PMC11677920; doi:10.3390/molecules29245882)
Supplement: Supplementary file 1 [file molecules-29-05882-s001.zip › molecules-3331722-supplementary.pdf]

Supplementary Material

For

Molecular design and mechanism study of collectors for sphalerite (ZnS) based on coordination chemistry theory and quantum chemical simulation

Xiaoqin Tang [1], Yilang Pan [1], Jianhua Chen [1,2,*], Ye Chen [1,2,*]

1. School of Chemistry and Chemical Engineering, School of Resources, Environment and Materials, State Key Laboratory of Featured Metal Materials and Life-cycle Safety for Composite Structures, Guangxi University, Nanning 530004, China;

2. Guangxi Higher School Key Laboratory of Minerals Engineering, Guangxi University, Nanning 530004, China

1. Materials

Sphalerite adopted in the single mineral flotation tests was sourced from the Nei Monggol Autonomous Region, China. The X-ray diffraction (XRD) tests of sphalerite samples are shown in Figure S1. The bulk minerals were ground and then screened to acquire samples with grain sizes ranging from 38 to 76 μm for flotation tests. Collectors BX and 1,2-BDT, activator $CuSO_4$, frother methyl isobutyl carbinol (MIBC) and pH modifiers sodium hydroxide (NaOH) and sulfuric acid (H_2SO_4) were purchased from Florrea Chemical Co., LTD., Shenyang, China, with analytically pure. Other reagents adopted in the experiments were analytically pure reagents purchased from commercial sources.

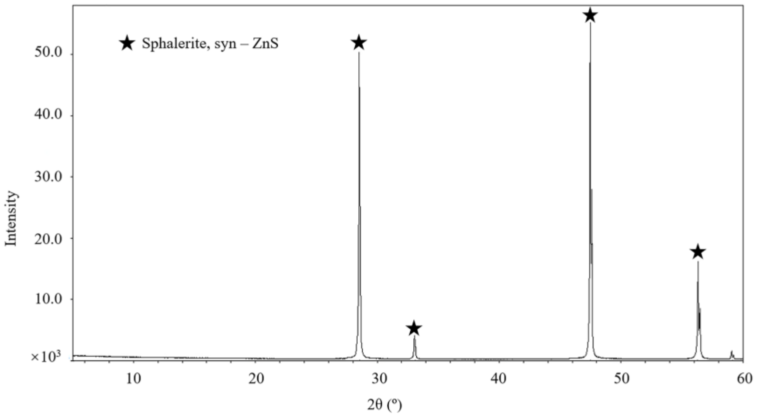

Figure S1 XRD analysis results of sphalerite

2. Single mineral flotation tests

Single mineral flotation tests were carried out by an XFGCII air-filled hanging tank flotation machine (Changchun Exploring Machinery Factory, China). The specific flotation process was as follows. Firstly, 2.0 g of mineral samples were immersed in a

beaker containing 10 mL deionized (DI) water for ultrasonic cleaning to remove the surface oxides, and then dispersed into a 40.0 mL flotation cell. Secondly, the pH modifier, activator, collector and frother were added in sequence and the corresponding stirring time for each reagent was 2 mins, 2 mins, 4 mins and 2 mins. The collection time for concentrates was 5 mins. The impeller speed was set to 1740 r/min. DI water was always used in reagent preparation and flotation tests. The recovery is calculated on the basis of the weight of dried concentrates and tailings. The calculation formula is shown in Eq. S1.

$$R = \frac{m_c}{m_c + m_t} \times 100\% \tag{S1}$$

Where R is the recovery (%), m_c represents the mass of the concentrates (g), and m_t denotes the mass of the tailings (g).
